# Wind-driven and buoyancy-driven circulation in the subtropical North Atlantic Ocean

**DOI:** 10.1098/rspa.2021.0172

**Published:** 2021-12

**Authors:** Harry L. Bryden

**Affiliations:** School of Ocean and Earth Science, University of Southampton, Empress Dock, Southampton SO14 3ZH, UK

**Keywords:** ocean circulation, wind-driven circulation, buoyancy-driven circulation, Atlantic Meridional overturning circulation, Gulf Stream, Rapid observations

## Abstract

Continuous observations of ocean circulation at 26°N in the subtropical Atlantic Ocean have been made since April 2004 to quantify the strength and variability in the Atlantic Meridional overturning circulation (AMOC), in which warm, upper waters flow northward and colder deep waters below 1100 m depth return southward. The principal components of the AMOC are northward western boundary current transport in the Gulf Stream and Antilles Current, northward surface Ekman transport and southward thermocline recirculation, all of which are generally considered to be part of the wind-driven circulation. Southward flowing deep waters below 1100 m depth are usually considered to represent the buoyancy-driven circulation. We argue that the Gulf Stream is partially wind-driven but also partially buoyancy-driven as it returns upper waters upwelled in the global ocean back to water mass formation regions in the northern Atlantic. Seasonal to interannual variations in the circulation at 26°N are principally wind-driven. Variability in the buoyancy-driven circulation occurred in a sharp reduction in 2009 in the southward flow of Lower North Atlantic Deep Water when its transport decreased by 30% from pre-2009 values. Over the 14-year observational period from 2004 to 2018, the AMOC declined by 2.4 Sv from 18.3 to 15.9 Sv.

## 1. Introduction

With the end of the official decade of the World Ocean Circulation Experiment (WOCE) in 2000, oceanography changed from a science intent on measuring the ocean circulation globally for the first time to a science trying to understand what sets the strength of the ocean circulation and how the circulation will change in a changing climate.

**Figure 1.**
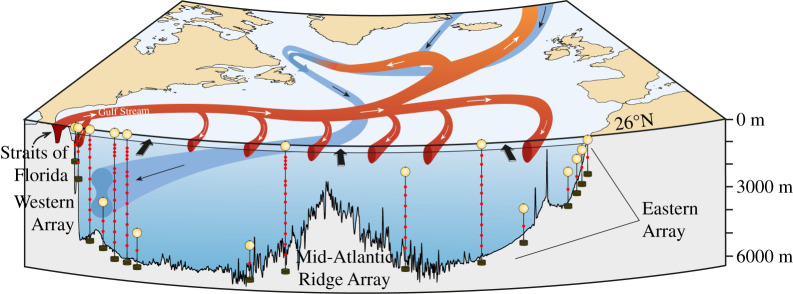
Rapid instrument array along with 26°N superimposed on schematic of the circulation of the North Atlantic Ocean including Gulf Stream flow through Florida Straits, Antilles Current just east of the Bahamas, DWBC with two cores and mid-ocean thermocline recirculation. Black arrows at the surface indicate wind-driven Ekman transport.

There has been an intense effort since 2004 to measure the variability in the basin-scale Atlantic Ocean circulation. A key motivation for this effort is to quantify the size of the Atlantic Meridional overturning circulation (AMOC), in which warm upper waters flow northward, lose heat to become cold deep water that then flow back southward at depths below 1100 m. The AMOC is a persistent feature of Atlantic Ocean circulation from its southern boundary at 30°S as far north as 70°N. Many monitoring efforts have been mounted to measure components of the AMOC, as detailed by Frajka-Williams *et al.* [1]. We concentrate here on the AMOC observations at 26°N [2] because they are the longest basin-scale monitoring system in duration and a key issue is to assess whether the AMOC is declining. Figure 1 is a schematic of the AMOC with the monitoring system of moored instruments deployed along 26°N since 2004. At a latitude of 26°N, the principal northward flows are in the Gulf Stream through Florida Straits above 800 m depth and Antilles Current in a 45 km zone east of the Bahama Islands also above 800 m depth and in the mid-ocean wind-driven surface Ekman layer. The principal southward flows are in the deep western boundary currents (DWBCs) between 1100 and 5000 m depth in a 150 km wide zone just east of the Bahamas [3] and in the mid-ocean geostrophic thermocline flow from the surface to 800 m depth. The southward mid-ocean thermocline flow is generally considered to recirculate northward in the western boundary currents. Overall across 26°N, there is a net northward flow above 1100 m depth and a net southward flow below 1100 m depth, and the net northward flow provides the definition for the strength of the AMOC.

The ocean circulation at 26°N transports heat northward at a rate of about 1.25 PW [4] and freshwater southward at a rate of about 0.5 Sv [5]. Nearly all of the heat and freshwater transports are due to the AMOC with horizontal recirculations accounting for only about 10% of the total heat or freshwater transports. Hence temporal changes in the AMOC can be linked to changes in the distribution of heat and salt content in the ocean north of 26°N and south of 26°N [6,7]. Variations in strength of the AMOC on paleo time scales have been linked to millennial changes in climate like the ice ages [8].

Following the WOCE, there were estimates for the strength of the AMOC based on synoptic transocean hydrographic ship surveys, in which the AMOC peaked at about 18 Sv at 25°N in the Atlantic [9]. These hydrographic surveys provided the detailed distribution of temperature, salinity and velocity along with the mid-ocean section so that the overall water mass transformations could be explored in temperature classes [10]. From six hydrographic sections along 25°N from 1957 to 2010, there was a consistent northward flow of surface waters (with T > 22.5°C), lower thermocline waters (9.5–12.5°C) and intermediate waters (5–9.5°C) with recirculation of main thermocline waters (12.5–22.5°C) flowing southward in mid-ocean and returning north in the Gulf Stream. And there was consistent southward flow in all deep waters separated into temperature classes 1.8–2.5–3.2–4–5°C. Thus, the overall transformation was from warm surface waters warmer than 22.5°C and lower thermocline and intermediate waters with temperatures between 5° and 12.5°C into cold deep waters with temperatures of 1.8 to 5°C. There was little information, however, on how accurate these synoptic sections values were and how the AMOC varies on seasonal, interannual and decadal time scales.

The AMOC at 26°N represents the net effects of the water mass transformation processes that occur in the northern Atlantic that transform about 18 Sv of northward flowing warm, salty upper waters into 18 Sv of cold, fresher southward flowing deep waters [9]. While a simplistic view is often that this transformation is linked to deep water formation in the Labrador and Norwegian Seas, Xu *et al*. [11] describe the complex water mass transformation processes that ultimately lead to deep water formation and its return back southward. The AMOC at 26°N also represents a summary of some water mass transformation processes that occur in the global ocean that convert 18 Sv of cold, relatively fresh southward flowing deep water into 18 Sv of warm, saltier upper waters returning to the northern Atlantic. A key process transforming deep water into upper waters is the upwelling of deep waters across the Antarctic Circumpolar Current [12].

The UK-US Rapid-MOCHA-WBTS (Meridional Overturning Circulation Heatflux Array-Western Boundary Time Series) project sets out to continuously measure the AMOC at 26°N and has made 14 years of observations from March 2004 to February 2018. A recent update (July 2021) has extended the time series up to February 2020. Coronavirus issues delayed the recovery and redeployment of instruments at 26°N deployed in 2018 and the continuation of Rapid measurements beyond 2022 is uncertain. Thus, it is appropriate to review what we have learned about the basin-scale Atlantic Ocean circulation and its variability over the 14 years 2004–2018.

There have been several recent reviews of Atlantic Ocean circulation including the AMOC notably by Buckley & Marshall [13], Frajka-Williams *et al*. [1] and Srokosz *et al*. [14]. Here we concentrate first on what the Rapid observations teach us about the wind-driven and buoyancy-driven ocean circulation at 26°N near the centre of the Atlantic subtropical gyre. Proxy indices suggest that the AMOC may have declined over the past century [15] and coupled ocean-atmosphere climate models project a significant decline in the AMOC over the next 50 years [16]. Thus, an important issue is to assess whether the Rapid observations over 14 years exhibit a decline in the AMOC.

## 2. Observational framework

**Figure 2.**
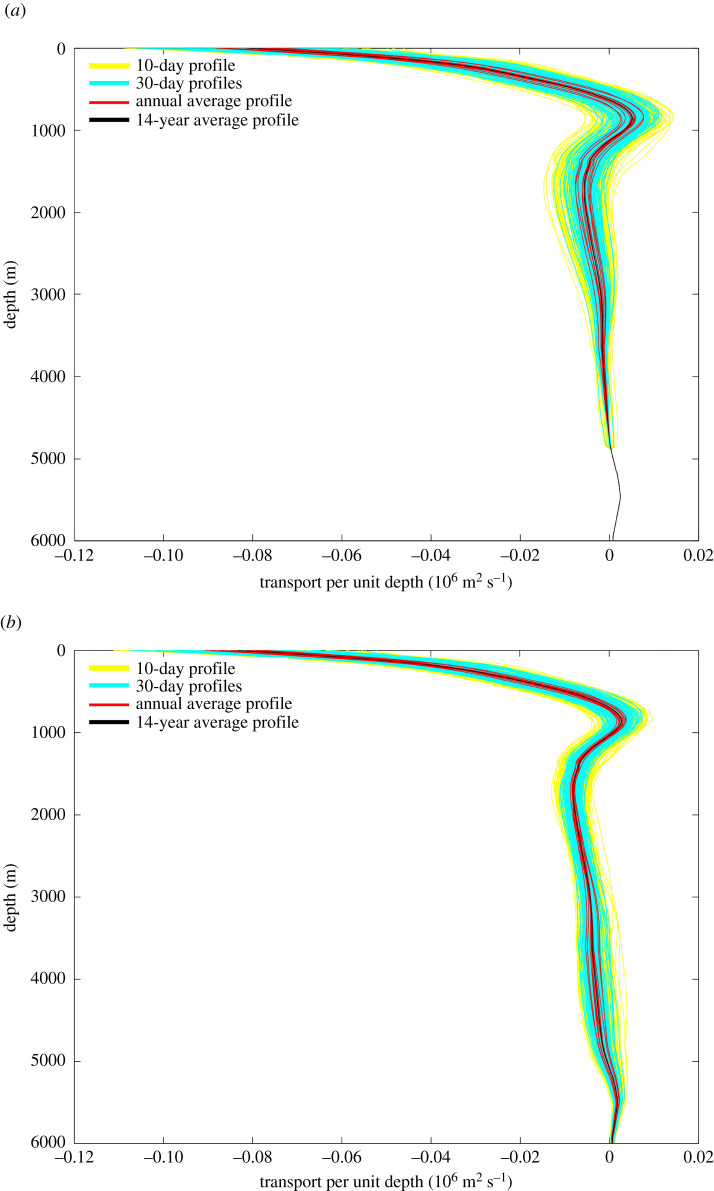
(*a*) Geostrophic velocity profiles referenced to 4800 dbar derived by differencing dynamic height profiles between the eastern and western Rapid moorings. (*b*) Geostrophic velocity profiles with reference level velocity at 4800 dbar chosen so that the northward mass transport across 26°N equals 0 at each time.

In 2002, it was uncertain whether the AMOC could be measured reliably. Science funding agencies NERC, NSF and NOAA agreed to support the deployment and analysis of a pilot Rapid array to determine whether the AMOC could be monitored continuously. The location for the monitoring effort was chosen to be 26°N to build on the long time series of Gulf Stream transport through the Straits of Florida. At 26°N, the principal northward flow of the Gulf Stream has long been monitored by a submarine electromagnetic cable across Florida Straits between Florida and the Bahamas [17]. The principal problem for Rapid then was to measure continuously the top-to-bottom mid-ocean flow between the Bahamas and Africa. To do this, deep water moorings are deployed on the western and eastern edges of the 26°N section (figure 1). These moorings are instrumented principally with sensors measuring temperature, conductivity and pressure at many levels from near the surface to just above the bottom so that on each side of the ocean a time series of density profiles is obtained. Differencing these eastern and western density profiles and using the thermal wind relations produce time series for the vertical shear in zonally averaged meridional geostrophic velocity that are then integrated vertically to yield time series of zonally averaged mid-ocean geostrophic velocity profiles referenced to a deep reference level (figure 2*a*). Rapid chooses 4800 dbar (4720 m) for the reference level. Wind-driven Ekman transports in the upper 100 m are determined from satellite-based wind stress measurements on a continuing basis. That the sum of the Gulf Stream, Ekman and mid-ocean geostrophic transport referenced to 4800 dbar does not equal zero has been known for many years due to an overall southward velocity at the reference level [18]. Because the North Atlantic basin north of 26°N is nearly closed (only a small flow from the Pacific through Bering Straits enters at the northern boundary of the Atlantic), Rapid methodology effectively sets the reference level velocity for the mid-ocean geostrophic velocity profile (figure 2*b*) so that the total northward Gulf Stream + Ekman + mid-ocean geostrophic transport across 26°N equals zero at each time. McCarthy *et al*. [19] describe the methodology in greater detail.

**Figure 3.**
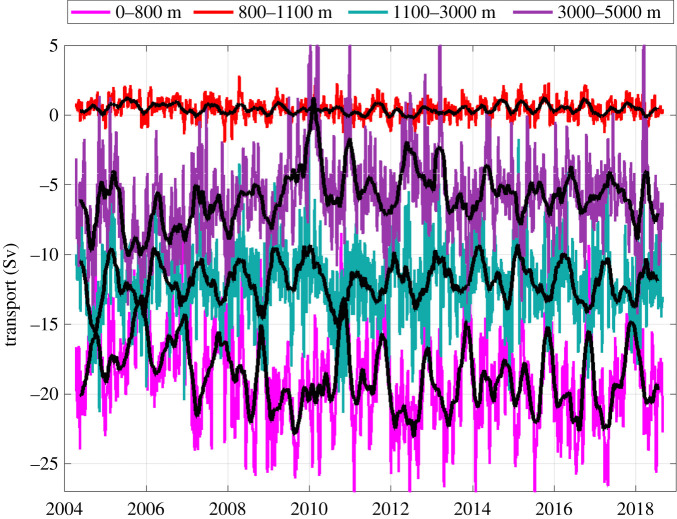
Time series of mid-ocean layer transports derived from figure 2*b* for 0–800 m (Thermocline Recirculation), 800–1100 m (AAIW), 1100–3000 m (UNADW) and 3000–5000 m depth (LNADW). The standard figure can be obtained from the Rapid website.

**Figure 4.**
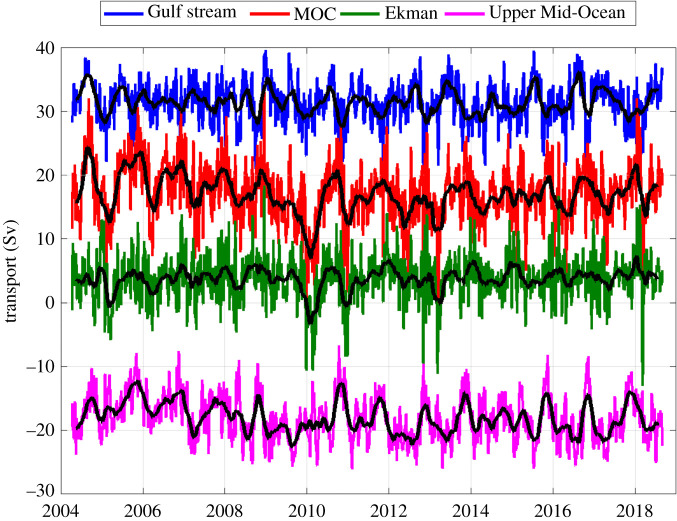
Time series of Gulf Stream, Ekman and Upper Mid-Ocean transports and their sum representing the Meridional overturning circulation at 26°N. The standard figure can be obtained from the Rapid website.

There are nuances to the above method as used by Rapid including (i) direct current measurements in the western boundary wedge region between the Bahamas and the westernmost hydrographic mooring 10 km offshore; (ii) deep hydrographic moorings on the western and eastern sides of the Mid Atlantic Ridge below 3000 m depth so that the mid-ocean geostrophic velocity can be separated into western and eastern basin contributions; and (iii) bottom pressure gauges to measure temporal variability in the reference level velocity [19]. Overall, the ocean circulation measured within the Rapid framework has three components: a shallow wind-driven Ekman transport in the upper 100 m (northward on average), a Gulf Stream transport through Florida Straits above 800 m depth (northward) and a top-to-bottom profile of mid-ocean geostrophic transport (southward overall). The mid-ocean geostrophic transport (figure 2*b*) is commonly separated into layers (figure 3): southward geostrophic flow from 0 to 800 m depth called thermocline recirculation here, northward flow from 800 to 1100 m depth called Antarctic Intermediate Water (AAIW), southward flow from 1100 to 3000 m called Upper North Atlantic Deep Water (UNADW) with origins in the Labrador and Irminger Seas, and southward flow from 3000 to 5000 m called Lower North Atlantic Deep Water (LNADW) with origins in the Nordic Seas. Below 5000 m, there is a small northward flow of Antarctic Bottom Water that exhibits only small variability [20]. To define the strength of the AMOC, Rapid commonly displays time series for Gulf Stream, Ekman and Mid-ocean Upper Geostrophic (0 to 1100 m depth) transports and their sum to represent the maximum northward flow of upper ocean waters (figure 4).

The Rapid observational strategy effectively estimates bulk transformations in depth coordinates where upper waters are turned into deep waters. The strategy is based on the powerful spatial averaging inherent in geostrophic dynamics where the depth profile of zonally averaged basin-scale meridional flow can be determined from endpoint measurements. An alternative approach would be to estimate flow in density layers so that subtle changes in temperature and salinity within density layers could be monitored in addition to the bulk transformations from lower density upper waters into higher density deep waters. Such an approach requires observations across the basin to measure the topography and water mass characteristics of the density layers. Without comprehensive observations across the basin and in the Gulf Stream, e.g. continuous hydrographic sections, this alternative approach cannot provide the accurate estimates of water mass transformations needed to assess whether the AMOC is changing over time.

Each of the major tenets underlying the Rapid estimates of AMOC variability has been challenged and investigated. Do the temporal variations in reference level velocities truly exist? Kanzow *et al*. [21] used bottom pressure measurements to show that geostrophic bottom velocity variations implied by the bottom pressure gradients indeed matched the reference level velocity variations for periods of 10 days to a year. Do temporal variations of boundary dynamic height due to eddies dwarf the basin-scale signal [22]? Kanzow *et al*. [23] showed that eddy variability decreases markedly as eddies interact with the western boundary and lose energy so the zonally averaged mid-ocean velocity signal associated with the basin-wide slope of the thermocline remains larger than the eddy variability at the boundary. Are the currents in the energetic western boundary region ageostrophic as asserted by Stepanov *et al*. [24]? Careful geostrophic comparisons both with observations and in models [25] showed that even in the strong currents near the Bahamas, the dynamical balance remains geostrophic for the meridional velocities. Thus, the Rapid estimates of AMOC structure and variability have remained robust. McCarthy *et al*. [19] carefully described the methodology used in Rapid to measure the AMOC components and estimated the overall accuracy of the AMOC transports to be 1.5 Sv on 10-day time scales and 0.9 Sv on annual averages.

## 3. Wind-driven and buoyancy-driven ocean circulation

**Figure 5.**
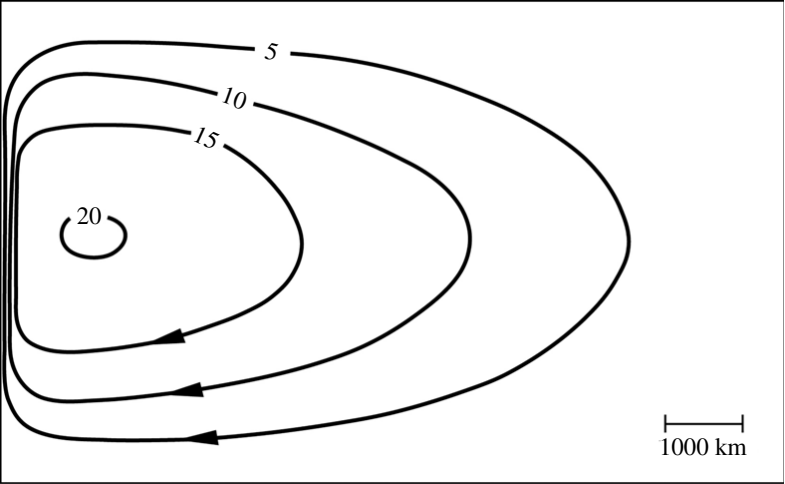
Modified version of Stommel's [27] model for the wind-driven circulation in a subtropical gyre for where the zonal wind stress varies with latitude as a cosine with Trade Winds in the south and Westerlies to the north and where Coriolis force varies linearly with latitude. Here the original wind stress forcing is reduced by a factor of two for better agreement with wind stress climatologies at 26°N. The resulting circulation is in reasonable agreement with the observed strength of the mid-ocean thermocline circulation and with a wind-driven western boundary current transport of 19 Sv at 26°N.

Ocean circulation is commonly considered to be a combination of wind-driven and buoyancy-driven circulations. Sometimes the buoyancy-driven circulation is called the thermohaline circulation although Wunsch [26] argued persuasively that thermohaline circulation is a poorly defined term. A fundamental understanding of both wind-driven and buoyancy-driven circulations is due to Stommel. His theory for western intensification (figure 5, [27]) showed that the wind stress distribution over a subtropical gyre like that in the North Atlantic with Westerlies to the north and Trade Winds to the south forces convergent flow in the wind-driven surface Ekman layer and equatorward geostrophic mid-ocean flow beneath the Ekman layer that must be balanced by a strong poleward flow in a western boundary current like the Gulf Stream. The theory is quantitative in that the wind stress drives a surface Ekman transport, the curl of the wind stress over a subtropical gyre drives equatorward geostrophic Sverdrup transport and the western boundary current transport equals the sum of Ekman and geostrophic Sverdrup transports.

**Figure 6.**
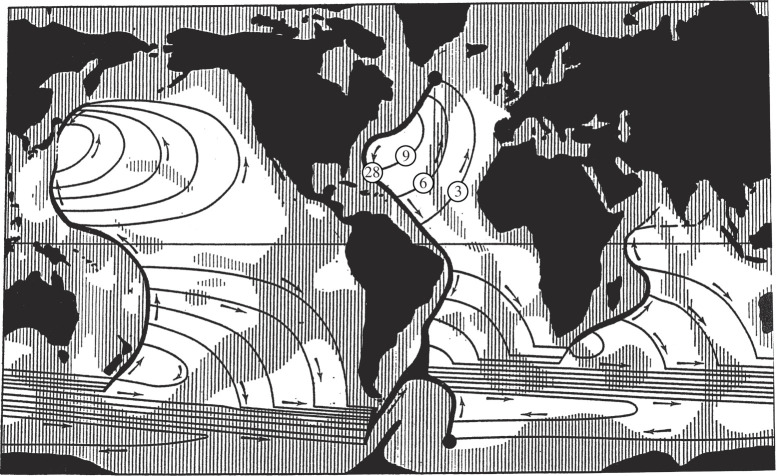
Stommel's [28] schematic of the buoyancy-driven abyssal circulation where deep water formation of 20 Sv occurs in the northern Atlantic and 20 Sv in the Weddell Sea and upwelling out of the abyss occurs uniformly over the world ocean. Rapid observations are indicated for a mean deep western boundary transport of 28 Sv at 26°N with 9 Sv of deep water recirculating northward in mid-ocean so that an overall southward deep water transport of 18 Sv across 26°N is identified.

Stommel's [28] model for the buoyancy-driven circulation starts with an assumption of deep water formation in the northern Atlantic and Weddell Sea based loosely on observed deep water properties. He then assumes a uniform upwelling of the deep water over the global ocean and this upwelling drives poleward mid-ocean flow of deep water everywhere. The deep water circulation is then closed with strong DWBCs in each ocean basin (figure 6). In the North Atlantic, the DWBC is directed equatorward away from the deep water formation region in the northern Atlantic. To complete the buoyancy-driven circulation, there must be a return flow of upper waters back toward the deep water formation region and arguably that return flow is in the western boundary current. Thus in the North Atlantic, the Gulf Steam includes both wind-driven and buoyancy-driven components. Stommel's model for the buoyancy-driven circulation is not quantitative in the sense that the size of deep water formation is assumed to be 20 Sv in the northern Atlantic and 20 Sv in the Weddell Sea, and to date there is no widely accepted theory for what sets the size of the deep water formation and the strength of the buoyancy-driven circulation [29].

The Rapid project has endeavoured to measure all components of the wind-driven and buoyancy-driven ocean circulation at 26°N near the centre of the subtropical gyre in the North Atlantic Ocean and to quantify the temporal variability in each of the components. We interpret the Rapid observations in the framework of the wind-driven and buoyancy-driven circulations defined by Stommel [27,28]. We start with the time-averaged circulation as measured within Rapid and then examine the 10-day, monthly, seasonal and interannual variability in each component. First, we describe the upper water flows associated with the wind-driven circulation.

## 4. Upper water circulation

For the circulation in the upper 800 m, Rapid observations (figure 3) yield a 14-year time-averaged Gulf Stream transport through Florida Straits of 31.4 Sv, a northward surface Ekman transport of 3.7 Sv and a southward mid-ocean geostrophic transport above 800 m depth of 18.8 Sv (table 1). East of the Bahamas, there is the northward flowing Antilles Current in the upper 800 m with a time-averaged transport of 4.7 Sv [30]. The Rapid methodology using end station profiles effectively includes this Antilles Current transport in the mid-ocean 0–800 m geostrophic transport. Here, we consider the Antilles Current to be part of the western boundary current. The time-averaged northward flowing western boundary current transport is then 36.1 Sv with 31.4 Sv flowing through Florida Straits and 4.7 Sv in the Antilles Current just east of the Bahamas. Removing the northward flowing Antilles Current just east of the Bahamas (4.7 Sv) from the overall 18.8 Sv southward 0 to 800 m geostrophic transport between the Bahamas and Africa results in a total southward mid-ocean thermocline recirculation of 23.5 Sv. These are effectively the same components as those in wind-driven ocean circulation theory: surface wind-driven Ekman transport, mid-ocean geostrophic thermocline recirculation driven by the curl of the wind stress and a western boundary current providing mass balance. In the Sverdrup theory, the western boundary current balances the sum of the Ekman and mid-ocean thermocline recirculation transports. From Rapid observations, however, the western boundary current transport of 36.1 Sv is 16.3 Sv larger than the sum of the Ekman and thermocline recirculation transports of 19.8 Sv.

**Table 1.** Statistics for AMOC components. Moored Rapid observations at 12-hourly intervals from which these values are calculated extend from 2 April 2004 to 3 September 2018. Ten-day values are calculated over 21 12-hourly values; monthly values are calculated over 61 12-hourly values; yearly values are calculated over 731 12-hourly values.

		standard deviation		
	mean	10-day	monthly	yearly
Gulf Stream	31.42	2.84	2.36	0.59
Ekman	3.72	3.02	2.23	0.75
UMO (0–1100)	−18.35	3.46	3.04	1.32
GS + Ek + UMO	16.79	4.22	3.48	1.91
UGEO (0–800)	−18.76	3.19	2.85	1.23
AAIW (800–1100)	0.41	0.59	0.45	0.14
UNADW (1100–3000)	−11.93	2.29	1.72	0.48
LNADW (3000–5000)	−5.88	2.75	2.28	1.44

There is a small additional northward mid-ocean transport of AAIW between 800 and 1100 m depth of 0.4 Sv that we consider to be part of the buoyancy-driven circulation returning upwelled deep water from the global ocean back to the deep water formation region in the northern Atlantic. Overall at 26°N, there is a net northward transport above 1100 m depth of 16.8 Sv. This sum of Gulf Stream, Ekman and mid-ocean transports above 1100 m depth is used within Rapid effectively to define the strength of the Atlantic meridional overturning circulation.

It is clear from the Rapid observations that at 26°N the western boundary current transport is larger than the southward mid-ocean geostrophic transport in the upper 800 m. Based on wind-driven theory, the total depth-integrated meridional transport equals $\left(\partial \tau^{y}/\partial x-\partial \tau^{x}/\partial y)/(\rho \beta \right)$, often called the Sverdrup transport, where $\partial \tau^{y}/\partial x-\partial \tau^{x}/\partial y$ represents the curl of the wind stress *τ*, *β* is the northward gradient of the Coriolis parameter and *ρ* is the density of seawater. This total meridional transport is a combination of the wind-driven Ekman transport in the surface layer $-\tau^{x}/(\rho f)$ and geostrophic flow throughout the water column $\left(\partial \tau^{y}/\partial x-\partial \tau^{x}/\partial y)/(\rho \beta )+\tau^{x}/(\rho f\right)$ that is often called the geostrophic Sverdrup transport. In wind-driven theory, when the Ekman transport, $\tau^{x}/(\rho f)$, is added to the geostrophic Sverdrup transport, the wind-driven western boundary current transport should equal the Sverdrup transport, $\left(\partial \tau^{y}/\partial x-\partial \tau^{x}/\partial y)/(\rho \beta \right)$. Thomas *et al*. [31] examined the applicability of the Sverdrup balance within a coupled climate model and found that the Sverdrup transport provides a good representation of the southward flow vertically integrated from the surface to a depth of no motion at 1500 m in mid-ocean regions of the subtropical gyre outside the western boundary regions. The model western boundary was very wide, however, possibly due to the coarse resolution of the model.

The geostrophic Sverdrup transport near 26°N has been estimated by various authors from wind stress climatologies to be 21 to 25 Sv (25 Sv by Leetmaa *et al*. [32] from Leetmaa & Bunker [33] winds; 25 Sv by Longworth [34] from NOC winds; 23.7 Sv by Atkinson *et al.* [35] from NCEP winds; and 21 Sv (ERA-20C winds) or 25 Sv (NOAA-20CR winds) by Piecuch [36]). These estimates of geostrophic Sverdrup transport are in good agreement with the size of the 0–800 m thermocline recirculation of 23.5 Sv determined from the Rapid observations. We conclude that the observed thermocline recirculation at 26°N is equal to the geostrophic Sverdrup transport driven by the curl of the wind stress and the observed western boundary current transport of 36.1 Sv is more than 16 Sv larger than the western boundary transport predicted by wind-driven theory.

The western boundary current transport at 26°N, primarily through Florida Straits, is larger than predicted by wind-driven circulation theory. As argued by Schmitz *et al*. [37], the extra western boundary current transport of about 16 Sv is associated with the buoyancy-driven circulation, it is effectively the return surface flow required to supply the formation of NADW in the northern Atlantic. Much of this extra transport comes into the North Atlantic across the equator from the South Atlantic. In a mean salinity section for Florida Straits, Xu *et al*. [38] noted the clear evidence for South Atlantic components in the relatively low salinity waters near the surface and at depth. Schmitz & Richardson [39] estimated that 45% (about 14 Sv) of the flow through Florida Straits had come from the South Atlantic across the equator. In addition, there is about 1 Sv of AAIW flowing northward between 800 and 1100 m depth in the mid-ocean section at 26°N. These are return flows from the global ocean where deep waters have upwelled into the upper, near-surface waters and are returning to the source region of NADW formation. Overall, from Rapid observations, we conclude that the western boundary current transport of 36.1 Sv consisting of Gulf Stream flow through Florida Straits (31.4 Sv) and northward flow of the Antilles Current (4.7 Sv) is a combination of a 20 Sv wind-driven component and 16 Sv buoyancy-driven component.

## 5. Deep water circulation

At 26°N, the latitude of the Rapid observations, the DWBC is a strong narrow southward flow below 1100 m depth. In the mean, it is confined within 150 km of the western boundary defined by the Bahamas. From moored current metre observations, Biló & Johns [3] estimated a time-averaged southward transport of 28.3 Sv in the boundary current; 60% (17 Sv) of this transport is in the UNADW between 1100 and 3000 m depth and 40% (11 Sv) in the LNADW between 3000 and 5000 m depth. Offshore beyond 150 km from the Bahamas, there is northward recirculation of the deep waters. The zonal extent of this recirculation is uncertain. From available current metre moorings, Bryden *et al*. [40] estimated a northward recirculation of 13 Sv from 160 to 580 km offshore. Biló & Johns [3] used Argo profiles plus shipboard CTD and LADCP profiles to suggest a northward recirculation of 8 Sv between 150 and 500 km offshore with much of the recirculation just to the east of the boundary current in the deep Abaco gyre. Because of the uncertainty in the zonal extent of the recirculation, the overall southward transport of UNADW and LNADW across the 26°N section is not well defined by near-boundary observations.

The Rapid array observations that span the Atlantic basin at 26°N define a time series for the zonally averaged vertical profile of the southward mid-ocean flow across the entire 26°N section from the Bahamas to Africa (figure 2*b*, figure 3). The net southward transport below 1100 m depth averages 12 Sv for UNADW (1100 to 3000 m depth) and 6 Sv for LNADW (3000 to 5000 m depth). These net southward transports are each about 5 Sv smaller than the near-boundary DWBC transports estimated by Biló & Johns [3] suggesting a northward mid-ocean recirculation of deep water of about 10 Sv. The Rapid end stations estimates do not define the zonal extent of the recirculation.

Stommel's original model for the buoyancy-driven abyssal circulation was based on a 20 Sv injection of NADW in the northern Atlantic. His DWBC increased in transport from 20 Sv at source to roughly 24 Sv of southward flow at 30°N as the uniform upwelling out of the deep water over the Atlantic drives a uniform northward interior flow (deep recirculation) of about 6 Sv with 2 Sv being upwelled out of the deep water in the Atlantic north of 30°N [41]. The Rapid observations confirm a strong southward DWBC with northward recirculation in mid-ocean (figure 6). The observed DWBC of 28 Sv is larger than Stommel and Arons's 24 Sv and the recirculation of 10 Sv is larger than Stommel and Arons's 6 Sv but the order of magnitude agreement is fascinating given Stommel's simple assumptions of 20 Sv NADW injection in the northern Atlantic and uniform upwelling throughout the global ocean.

## 6. Temporal variations in wind-driven circulation at 26°N

All components of the basin-scale Atlantic circulation at 26°N are continuously observed by the Rapid array. We concentrate first on the subannual variations in the upper ocean components of the AMOC: Gulf Stream transport through Florida Straits, wind-driven surface Ekman transport, and southward mid-ocean geostrophic transport above 1100 m depth (figure 4). On 30-day and shorter time scales, there are variations in Florida Straits transport driven by local winds in the Straits [42] and by instabilities upstream in the Loop Current of the Gulf of Mexico [43]; variations in surface Ekman transport driven by winds associated with mid-ocean storms; and variations in upper ocean geostrophic transport associated with the slope of the thermocline across the Atlantic basin and with baroclinic eddies impinging on the boundaries [23]. These short-term variations in the AMOC surprised the Rapid investigators [2] in that previous estimates of the AMOC had been based on transatlantic hydrographic sections that captured the thermocline slope and that slope was considered to be representative of reasonably steady flow. The effect of eddies at the western boundary introduced variability in the overall thermocline slope so that the overall mid-ocean flow exhibits energetic variations on short time scales. Hence, hydrographic sections represent snapshots of a noisy signal. In fact, all estimates of AMOC transports from hydrographic sections are within the range of short-term variability observed in the first year of Rapid observations [2].

The temporal variations in 10-day average Gulf Stream transport, in Ekman transport and in mid-ocean thermocline transport above 800 m depth have standard deviations of 2.8, 3.0 and 3.2 Sv, respectively (table 1). Temporal variations in Ekman transport are not significantly correlated with Gulf Stream transport or with mid-ocean thermocline transport. As demonstrated by Frajka-Williams *et al*. [44], the short-term variations in Gulf Stream transport are negatively correlated with the mid-ocean thermocline transport above 800 m depth (−0.52 for 10-day values, −0.54 for monthly values) so there is some compensation between thermocline recirculation and Gulf Stream transport on short time scales. Overall, the standard deviation in AMOC transport (Gulf Stream + Ekman + Upper Mid-Ocean) is 4.2 Sv (10-day) or 3.5 Sv (30-day). If we assume monthly AMOC values are independent, then the uncertainty in annual-average AMOC is 1.0 Sv.

Ekman transport variations are negatively correlated with deep water transports on 10-day time scales (−0.46 for UNADW and −0.55 for LNADW), and this might partially be attributed to the Rapid methodology where variation in net upper transport (Gulf Stream + Ekman + upper mid-ocean transport) is compensated at each time by a nearly depth-independent flow, which is largely manifest in deep water transports. Neither Gulf Stream transport nor upper mid-ocean transport, however, is significantly correlated with deep water transports. Hence, variations in Ekman transport on short time scales do seem to be compensated by largely depth-independent flow, as suggested by Jayne & Marotzke [45], that are manifest in the variations in UNADW and LNADW transports with standard deviations of 1.7 and 2.3 Sv, respectively, for monthly averages.

There are small seasonal cycles in Gulf Stream and Ekman transports with amplitudes of 1.9 and 1.3 Sv respectively related to seasonal variations in the winds [46]. But the major seasonal variation is in the mid-ocean upper water circulation associated with variations in winds near the eastern boundary [47]. Wind stress curl variations drive a downwelling that depresses the depth of density surfaces as deep as 1000 m in September–October–November, leading to stronger northward mid-ocean flow in the waters above 1000 m depth [48]. This seasonal signal with an amplitude of 2.9 Sv and with maximum northward flow above 1100 m depth in October has also been explained in terms of seasonal changes in the poleward undercurrent and associated Mediterranean Water and AAIW flows [49], and it appears to be robust over 14 years of Rapid observations. It is confined to the region near the eastern boundary. Overall the seasonal signal in the AMOC defined by the sum of Gulf Stream, Ekman and upper mid-ocean flow above 1100 m depth has an amplitude of 2.5 Sv with maximum northward flow in October and is dominated by this eastern boundary downwelling [47]. This seasonal signal suggested that previous estimates of AMOC strength based on hydrographic sections (e.g. [9]) should be adjusted for the season in which the hydrographic sections were made. Such seasonal adjustments reduce the long-term decline in the AMOC suggested by previous analysis of historical hydrographic sections [48].

Using a two-layer model forced by observed winds over the subtropical Atlantic, Zhao & Johns [50,51] showed that wind-driven variations in the AMOC on seasonal to interannual time scales are caused by Rossby waves generated in mid-ocean that then propagate westward to affect the depth of the thermocline at the western boundary and hence the strength of the AMOC. Thus, variations in wind stress have been shown to cause variability in the AMOC and its components on subseasonal to interannual time scales.

## 7. Variations in the buoyancy-driven circulation at 26°N

**Figure 7.**
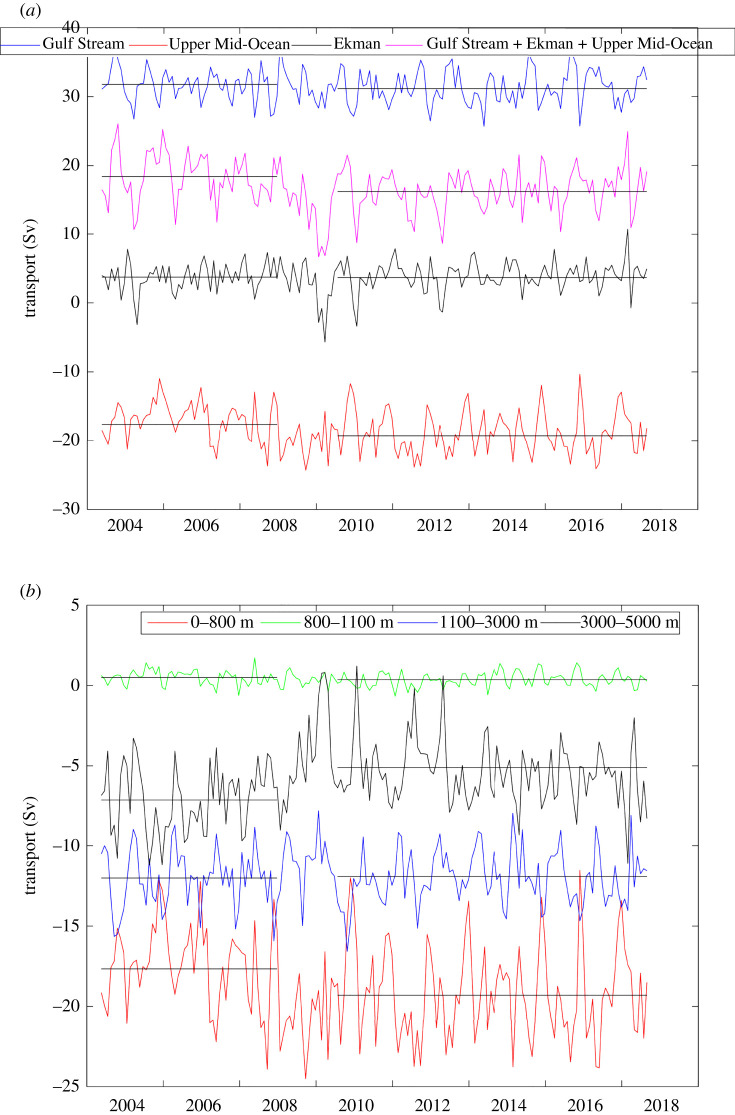
(*a*) Time series of upper water transport components for the 14-year duration of the Rapid observations. The sum of Gulf Stream + Ekman + Upper Mid-Ocean represents the AMOC. Averages for April 2004 to December 2008 and for July 2010 to July 2018 are shown as solid lines for each component and indicate the increase in thermocline recirculation and the decrease in the AMOC in early 2009. (*b*) Time series of mid-ocean layer transports for the 14-year duration of the Rapid observations. Averages for April 2004 to December 2008 and for July 2010 to July 2018 are shown as solid lines for each layer to indicate the increase in thermocline recirculation (0–800 m transport) and the decrease in LNADW (3000–5000 m) transport in early 2009.

With respect to the longer term variability in the AMOC and its components (figure 7), two significant changes over the 14-year observation period have been carefully documented: a sizeable reduction of nearly 5.7 Sv in the AMOC in 2009–2010 with recovery in 2011 [52] and a reduction of 2.4 Sv in 2008–2009 from an average AMOC of 18.3 Sv in the first 5 years of Rapid April 2004 to March 2009 to an average AMOC of 15.9 Sv for the period 2009 to 2018 [53]. A principal feature in both changes is a reduction in the southward transport of LNADW that is a component of the buoyancy-driven circulation. For the 2009–2010 event, the southward transport of LNADW decreased by 5.6 Sv from a mean value of 7.1 Sv before 2009 to near-zero transport in Summer 2010 (figure 7*b*). For the 2.4 Sv long-term reduction of the AMOC, the southward transport of LNADW decreased by 2.2 Sv from a mean of 7.1 Sv before 2009 to a mean of 4.8 Sv after 2009. There was no appreciable change in UNADW transport for either of these two significant changes.

For the 2009–2010 event, the decreasing LNADW transport was compensated initially in early 2009 by an increase in southward Thermocline Recirculation. Later, in Summer 2009, there was a decrease in northward Ekman transport and a decrease in northward Gulf Stream transport. Roberts *et al*. [54] attributed the compensating 5.7 Sv reduction in the upper northward flow of the AMOC to a 2.9 Sv increase in southward Thermocline Recirculation, a 1.7 Sv decrease in northward Ekman transport and a 1.1 Sv decrease in Gulf Stream transport. Overall, the decreased southward transport of LNADW during 2009–2010 was compensated by reduced northward flow in all three components of the upper water circulation. With the reduced overturning, northward heat and salinity transports across 26°N decrease, and there is cooling and freshening of the waters down to 1000 m depth north of 26°N through 2010 [55] and some warming and salinification of the waters south of 26°N [6] in reasonable quantitative agreement with the reduction in northward heat and salinity transports associated with the reduced AMOC.

For the long-term 2.4 Sv reduction in the AMOC, Smeed *et al*. [53] used change-point analysis to identify a step change in the AMOC in late 2008 or early 2009 that is possibly associated with the 2009–2010 event (figure 7*a*). In the deep water, the AMOC reduction was almost entirely due to a reduced southward transport of LNADW with no detectable change in UNADW transport (figure 7*b*). In terms of the compensating upper water components, the AMOC reduction was associated with a 0.6 smaller Gulf Stream flow through Florida Straits and a 1.7 Sv increase in southward Thermocline Recirculation after 2009. There was no change in Ekman transport associated with the long-term AMOC reduction. As a result of the reduced AMOC and reduced northward heat and salinity transports across 26°N, there has been a continuous cooling and freshening of waters in the subpolar North Atlantic above 800 m depth from 2009 to 2018 in reasonable quantitative agreement with the reduction in northward heat and salinity transports associated with the reduced AMOC [56], and recently, there has been a salinification in the South Atlantic that Zhu & Liu [7] attribute to the reduction in the AMOC.

**Figure 8.**
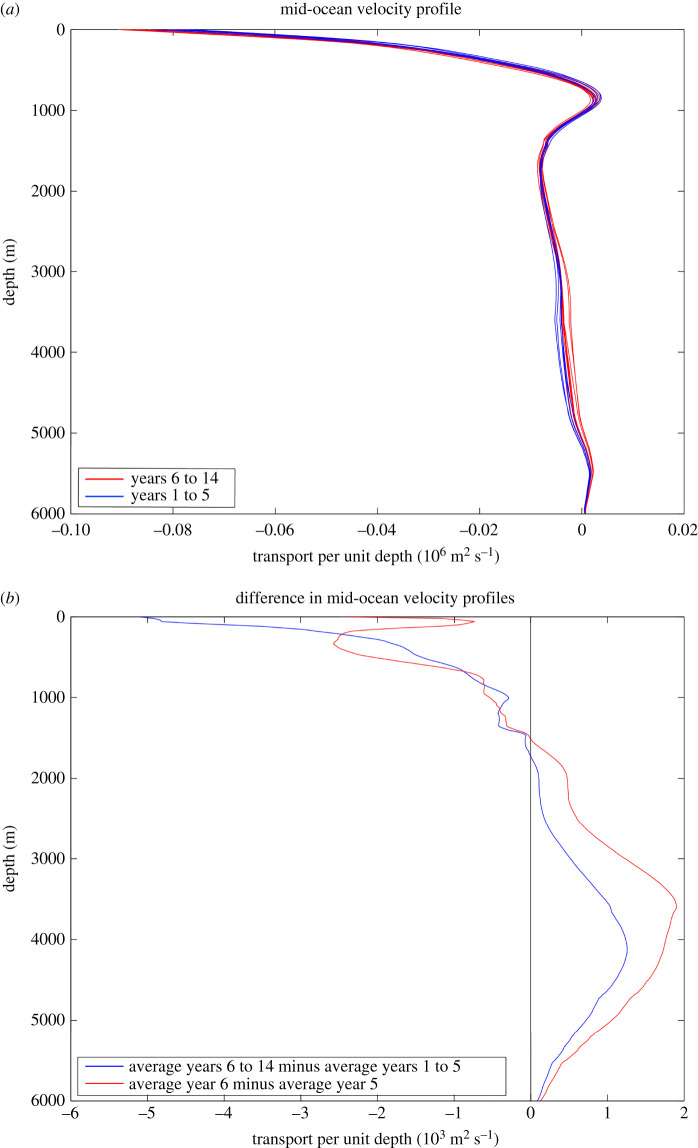
(*a*) Annual average profiles for zonally averaged mid-ocean northward velocity at 26°N. Blue profiles are for the first five years of Rapid observations April 2004 to March 2009. Red profiles are for the 9 years April 2009 to March 2018. (*b*) Profiles of the difference in annual-averaged mid-ocean northward velocity profile. Red is the difference between year 6 (April 2009 to March 2010) and year 5 (April 2008 to March 2009); blue is the difference between the average for years 6 to 14 (April 2009 to March 2018) and the average for years 1 to 5 (April 2004 to March 2009). The sharp reduction in LNADW transport (3000 to 5000 m) in early 2009 is evident in both. (Online version in colour.)

For both the 2009–2010 event and the long-term AMOC reduction, there is a marked change in the vertical structure of the mid-ocean flow. Annual-averaged profiles (figure 8*a*) show a reduction in the southward LNADW flow after 2008–2009. The difference between annually averaged profiles (figure 8*b*) exhibits a striking change in the deep water flow that is somewhat compensated by increased southward flow in the thermocline above 800 m depth. There is a change in the deep shear between UNADW and LNADW whereby the southward LNADW flow decreases relative to the nearly constant southward UNADW flow [44]. The signature in density for this change in shear is a deepening of isopycnals near the western boundary. Isopycnals below 1800 all deepen after 2009 with a maximum change in the horizontal density gradient and hence vertical shear near 3000 m depth [44]. The difference is magnified for the 2009–2010 event over the long-term change 2009–2018 minus 2004–2009 (figure 8*b*) but for both it is the deep shear between UNADW and LNADW that changes.

The time series of DWBC transport east of the Bahamas exhibits large variability [3] with a standard deviation of 17 Sv. These temporal variations are largely balanced by variations in the strength of the deep Abaco gyre. The magnitude of the temporal variability in the DWBC has so far made it impossible to detect the signature of small (order 2.5 Sv) changes in the southward transport of LNADW within the narrow boundary current.

In terms of the interannual variability in the components of the AMOC, there is evidence for significant temporal reductions in the southward flow of LNADW that is a principal component of the buoyancy-driven circulation in the North Atlantic. For mass balance, these reductions in southward deep water flow must be compensated by reductions in the net northward flow of warm, upper waters. For the two significant changes observed by the Rapid array since 2004, the upper water compensation has been mainly provided by a reduced southward thermocline recirculation with some reduction in northward Gulf Stream transport through Florida Straits.

## 8. Is the Atlantic Meridional overturning circulation declining?

Changes in the Atlantic meridional overturning circulation have been linked with paleoclimate changes: a weak overturning linked to cold ice ages, the modern strong overturning linked to equitable northern hemisphere Holocene climate with sharp temporal changes in the AMOC causing climate transitions [8]. Numerous paleoclimate proxies for the AMOC have been developed to construct long time series of the AMOC strength. Recent proxies that are based on sea surface temperature patterns and sediment patterns associated with deep currents [15,57,58] have indicated a long-term reduction in the AMOC since at least 1850. In many coupled climate models, an increase in atmospheric CO_2_ results in a slowdown in the AMOC and IPCC assessments have struggled to conclude whether increasing atmospheric CO_2_ will lead to a sizeable change in the AMOC [59–61]. In the past year, new CMIP6-coupled climate models all suggest an order of 50% reduction in the AMOC over the next 50 years [16]. Thus, a key question is whether the AMOC is presently in decline.

Over the Rapid observational period 2004 to 2018, the AMOC has indeed declined. It is not a steady decline, as can be seen in the time series of upper and deep water components (figure 7). It has happened in two possibly related steps, a 5.7 Sv decline in the AMOC during a 2009–2010 event with subsequent partial recovery [52] and a sharp reduction of 2.4 Sv in early 2009 reflecting a long-term reduction in the AMOC strength [53]. Both changes are associated with a reduction in southward transport of LNADW with no change in the transport of UNADW. For the upper waters, the decrease in the AMOC is due to a slightly reduced Gulf Stream transport and to more Gulf Stream waters recirculating southward in the subtropical gyre. Thus, the primary evidence for a declining AMOC at 26°N is the reduced southward flow of LNADW that is formed in the Nordic Seas.

Based on Rapid observations, the AMOC has declined by about 12% from 2004 to 2018. The decline occurred in a sharp reduction in early 2009 and not in a gradual, steady decline suggested by proxy time series. The proxies are often based on sea surface temperature records in the Atlantic and observed temperature changes may be related to global warming rather than changes in the AMOC [62]. In the subpolar gyre where temperature (and salinity) has steadily declined as a result of the reduced AMOC since 2009 [56], proxies based on surface temperature may capture accumulating effects of a change in the AMOC, but they do not reflect the sharp reduction in the strength of the AMOC.

Will the AMOC continue to decline? CMIP6 models suggest the AMOC will decline as a result of increasing atmospheric CO_2_ [16]. Others see oscillations in the AMOC with the recent decline a reaction to an earlier increase in the AMOC [63]. Re-analyses of ocean observations back to the 1980s indicate there has been little change in the AMOC over the past 30 years [64,65]. The Rapid team recently concluded that there are early signs for a rebound in the AMOC [66] but the updates to the Rapid time series through February 2020 suggests that the AMOC remains in a reduced state. The proxies argue for a long-term (century) decline in the AMOC that is unlikely to reverse. Rapid time series for the AMOC will soon be 18 years in duration following recovery of the presently deployed instrument array. Model-based estimates for when a long-term decline can be detected above the interannual variability in model AMOC strength concluded that a time series of several decades of AMOC variations is needed [67]. The Rapid AMOC time series will soon be long enough to test the reliability of proxy records and to compare with CMIP6 projections for the future strength of the AMOC.

## 9. Discussion

There has been a long debate as to whether the North Atlantic circulation including the Gulf Stream at about 25°N is in agreement with wind-driven Sverdrup transport [32,68]. Rapid observations show that the strength of the mid-ocean thermocline recirculation is in reasonable quantitative agreement with wind-driven geostrophic Sverdrup transport derived from available wind climatologies. However, the observed Gulf Stream transport through Florida Straits is substantially larger than that predicted for the western boundary current by wind-driven theory. The Gulf Stream and Antilles Current at 26°N should be considered to be a sum of the western boundary current that is predicted by wind-driven theory (20 Sv) and a buoyancy-driven western boundary current (16 Sv) that is returning upwelled surface and intermediate waters back to the deep water formation region in the northern Atlantic [37].

The Rapid observations indicate there has been a 2.4 Sv (30%) reduction in southward flow of LNADW across 26°N over the period 2004 to 2018. A substantial set of complementary moored observations of the DWBC near 37°N also concluded that the transport in LNADW decreased by 2.4 Sv over the 10-year period from 2004 to 2014 [69]. The reduction in southward flow of LNADW in the subtropical North Atlantic contrasts with observations that the Greenland-Scotland overflows have remained constant over the past 20 years [70,71], as these overflows represent the sources for the LNADW. If the LNADW inflow into the northern Atlantic has remained constant while the southward flow across 26°N (or 37°N) has reduced, then the reservoir of deep water between the Greenland-Scotland Ridge and 26°N would be filling. Bryden *et al*. [56] did find that the thermocline in the eastern subpolar gyre separating the deep and upper waters was shallowing by as much as 200 m over the period 2008 to 2018. They attributed this change to the reduction in northward flow of upper waters into the subpolar gyre, but such change is also consistent with a convergence of deep flows filling the subpolar reservoir of deep waters.

In this review, we concentrate on the AMOC observations at 26°N because they are the longest in duration and the AMOC at 26°N is relatively simple where much of the circulation can be efficiently monitored with boundary measurements. It is of interest to understand how the AMOC changes through the Atlantic and to assess whether the strength of the AMOC varies with latitude. A new project called OSNAP (Overturning in the Subpolar North Atlantic Program) has been monitoring the AMOC at about 55°N since 2014 [72]. As the warm upper waters progress from the subtropics into the subpolar gyre, the structure of the circulation becomes more complex. There are warm northward flowing waters near Scotland on the eastern boundary, with alternating strong northward and southward flowing currents in mid-ocean (North Atlantic Current, East Reykjanes Ridge Current, Irminger Current) and with additional boundary currents in the west (East Greenland Current flowing south, West Greenland Current flowing north, Labrador Current flowing south). All currents have deep and shallow components and all are being monitored by OSNAP to define the strength and structure of the AMOC in the subpolar gyre. These circulating waters are regularly subject to air-sea buoyancy exchanges including deep wintertime convection that make the waters progressively denser. As a result, warm, less dense waters flow north and cold, denser waters flow south at the same depth so a major part of the overturning is in density and not in depth. From careful analysis of measurements across the subpolar gyre, Li *et al.* [73] estimate the AMOC to average 16.6 Sv in density coordinates for the 4-year period 2014–2018 with no significant trend. Thus, the size of the subpolar AMOC appears to be about the same as the subtropical AMOC at 26°N. Observations of the AMOC at other latitudes are not as complete as those from the Rapid and OSNAP monitoring systems but ultimately we would like to define how the strength and structure of the AMOC vary throughout the Atlantic.

It is of interest to understand how the Atlantic circulation changes in coupled models that suggest a 50% decline in the AMOC over the next 50 years. In an analysis of a coupled climate model where CO_2_ was increased by 2% per year for 70 years, Thomas *et al*. [74] showed that the model deep water transport decreased by 5.3 Sv and this was balanced solely by a weakening of the northward surface western boundary current. Overall the model western boundary current transport decreased by 8.7 Sv where the additional 3.4 Sv could be explained by changing wind stress curl associated with weaker trade winds and westerlies reducing the southward mid-ocean Sverdrup transport. Thus, we might expect that reduced southward deep water flows will be ultimately balanced by changes in the component of the western boundary current returning upper waters from the global ocean back to the northern Atlantic.

Evidence for an AMOC decline in the upper water is confusing in that there has been only a small reduction in the northward flow of 0.6 Sv in the Gulf Stream during the past 14 years of the Rapid observational period. On longer time scales, there has been little change in Florida Straits transport over 40 years of monitoring by submarine cable, and over the past 150 years, there are indications from sea level trends for only a relatively small decrease in Gulf Stream transport by 1.7 Sv per century [36]. Thus, the reduction in Gulf Stream transport appears to be smaller than the change in LNADW transport. Within the Rapid observational period 2004–2018, the change in upper water circulation has been an increase in southward thermocline recirculation by 1.7 Sv where more of the Gulf Stream waters recirculate within the subtropical gyre and less Gulf Stream waters flow northward into the subpolar gyre to feed the deep water formation. Differences in wind stress climatologies [36] are too large to reliably assess whether the Sverdrup transport at 26°N has increased or decreased. If the southward wind-driven Sverdrup transport has indeed increased while the western boundary current transport remains constant, then the buoyancy-driven component of the Gulf Stream flow through Florida Straits may have decreased. Separating Gulf Stream flow through Florida Straits into recirculating North Atlantic waters (wind-driven) and low salinity waters of South Atlantic origin (buoyancy-driven) would help to identify the source of changes in the AMOC.

## Data accessibility

Data from the RAPID AMOC monitoring project is funded by the Natural Environment Research Council and are freely available from www.rapid.ac.uk/rapidmoc.
